# Evaluation of the Performance of CinTec® PLUS in SurePathTM Liquid-Based Cervico-Vaginal Samples

**DOI:** 10.5146/tjpath.2020.01505

**Published:** 2021-01-15

**Authors:** Pooja Sharma, Parikshaa Gupta, Nalini Gupta, Vanita Suri, Arvind Rajwanshi

**Affiliations:** Department of Cytology and Gynecological Pathology, Postgraduate Institute of Medical Education and Research, Chandigarh, India; Obstetrics and Gynecology, Postgraduate Institute of Medical Education and Research, Chandigarh, India

**Keywords:** Cervical cytology, Pap smear, Liquid-based cytology, CINtec PLUS, p16, Ki-67

## Abstract

***Objective:*** Cervical cytology and Human papillomavirus (HPV) testing are effective screening techniques but both have limitations. A few recent studies in the literature have highlighted the role of co-expression of p16INK4a and Ki-67 for cervical cancer screening. The present study was undertaken to evaluate the diagnostic performance of the CINtec® PLUS kit (dual immunostaining for p16 and Ki-67) in SurePathTM liquid-based (LBC) cervico-vaginal samples.

***Materials and Methods: *** This was a prospective study performed on 52 cervico-vaginal SurePath™ LBC samples reported as having squamous epithelial cell abnormality (ECA). All the samples were stained using CINtec® PLUS kits. Additionally, HPV-DNA testing was also done and the results were compared.

***Results: *** The age range was 34-74 years. ECA included 18 (34.6%) cases of ASC-US, 9 (17.3%) cases of low-grade squamous intraepithelial lesion (LSIL), 11 (21.2%) cases of high-grade squamous intraepithelial lesion (HSIL), and 14 (26.9%) cases of squamous cell carcinoma (SCC). Cervical biopsies were available in 19 (36.5%) cases. A total of 34/52 (65.4%) cases were positive for HPV-DNA (5/18-ASC-US; 6/9-LSIL; 10/11-HSIL; 13/14-SCC). The CINtec® PLUS test was positive in 41/52 (78.8%) cases (11/18-ASC-US; 6/9-LSIL; 11/11-HSIL; 13/14-SCC). On comparing CINtec® PLUS positivity (78.8%) with HPV positivity (65.4%), dual positivity was seen in 3/18 cases of ASC-US, 6/9 cases of LSIL, 10/11 cases of HSIL, and 12/14 cases of SCC. One case each of HSIL and SCC was negative on the HPV test and was positive on CINtec® PLUS.

***Conclusions: *** CINtec® PLUS test helps to improve the detection of pre-cancerous cervical lesions as compared to cervical cytology or HPV testing alone and hence can serve as a potentially useful diagnostic and triage tool, especially for indeterminate cases.

## INTRODUCTION

Cervical cancer screening using Papanicolaou-stained cervical smears is the most successful cancer screening program launched till date. The use of this screening method has lead to a significant increase in the detection rates of pre-cancerous lesions and a decrease in morbidity and mortality. Human papillomavirus (HPV) is the most common viral infection affecting the female genital tract and also the most important causative agent of cervical cancer. In the majority of the females, this viral infection is transient and self-limiting. However, in less than 10% of the cases, the virus persists and the epithelial cell abnormalities may progress to high-grade lesion or invasive cancer, which usually occurs over a period of several years (1). Several studies have demonstrated that HPV testing improves the sensitivity of detection of high-grade precursor lesions as compared to cervical cytology alone (2).

Cell cycle alterations induced by HPV oncoproteins during cervical neoplasia can serve as newer biomarkers that can be used to identify women who are at increased risk for developing cervical cancer precursors. Two such useful biomarkers are- p16INK4a, also known as p16 (a tumor suppressor protein), and Ki-67 (a cell proliferation marker) (3). Increased expression of p16INK4a and Ki-67 has been found to be associated significantly more commonly with high-grade cervical lesions (4). Thus, testing for these markers may provide enhanced specificity and diagnostic accuracy in HPV-positive women within an organised cervical screening programme.

The CINtec® PLUS test uses a dual immunostaining technique for detection of simultaneous p16INK4a and Ki-67 expression in the exfoliated cervical epithelial cells. This dual immunostaining kit reportedly helps to improve the detection of pre-cancerous cervical lesions. Only a few studies have assessed the utility of the CINtec® PLUS test on cervical samples, the majority of them being from developed countries of the world (5–7). There is paucity of data from the Indian subcontinent with only a few studies available (8). Therefore, the present study was undertaken to evaluate the diagnostic performance of CINtec® PLUS in SurePathTM liquid-based cervico-vaginal samples as compared to cervical cytology using the SurePath™ liquid-based cytology (LBC) technique and HPV testing performed with the Qiagen Hybrid Capture (HC2) assay and subsequent histopathology, wherever available.

## MATERIALS and METHODS

***Target Population***: Women aged 25 to 70 years, presenting to the gynaecology out-patient department with available Pap smears showing squamous epithelial cell abnormalities.

***Ethical approval:*** The study was approved by the institute’s ethics committee and a waiver of consent was granted (Reference No: NK/4102/Res, Date: 23.03.2018).

***Study type***: This was a prospective study, wherein a total of 52 cervico-vaginal samples, obtained during the study period (June 2017 to July 2018) and reported on cervical SurePath™ cytology as having squamous epithelial cell abnormality (as per The Bethesda System, 2014), were included. These samples were routinely screened by SurePath™ liquid-based cervical cytology in the Department of Cytology and Gynaecological Pathology, at a tertiary care centre. The samples collected in the SurePath™ preservative fluid collection vials were transferred to the concentration tubes, as is being done routinely for all SurePath™ cytology samples in the laboratory. All these cases were stained for dual immunostaining for p16 and Ki-67 using CINtec® PLUS kits on the samples from either the SurePath™ collection vials or the concentration tubes. HPV DNA testing was performed on these samples by using the Qiagen Hybrid Capture (HC2) assay.

***Sample processing from sample collection vials***: After the routine LBC smear preparation, the residual sample in the collection vial was equally divided into two parts: one was utilized for HPV testing and the second was used for performing the CINtec® PLUS test. The sample in the vial for CINtec® PLUS was vortexed, followed by centrifugation at 1500 rpm for 10 minutes. The supernatant was then discarded and the sediment resuspended in a tube, and this tube was then kept in the SurePathTM stainer for preparation of the LBC smears.

***Sample processing from concentration tubes***: After the routine LBC smear preparation, the residual material from the concentration tube was collected in a tube that was then used for LBC smear preparation using the SurePathTM stainer.

***CINtec® PLUS staining***: Prior to the staining procedure, the smears were rehydrated and treated with the Epitope retrieval solution followed by the staining procedure and aqueous mounting. CINtec® PLUS testing was performed manually as per the manufacturer’s protocol in all 52 cases. The test involves a two-step immunocytochemical staining procedure for the cytological specimens. For detection of the antigens, primary monoclonal antibodies are used and the chromogen reactions are based on the horseradish peroxidase-mediated conversion of 3, 3-diaminobenzidine (DAB) chromogen and alkaline phosphatase-mediated conversion of fast red chromogen to visible reaction products. The smears were prepared as per the routine SurePathTM protocol. Double immuno-reactive cells in smears indicate positive staining for both proteins. p16 shows nucleo-cytoplasmic positivity in the transformed cells. Ki-67, being a proliferative marker, stains the nucleus. Since DAB is used for p16 detection, p16 staining is seen as a brown color and similarly, as fast red is used for Ki-67 detection, Ki-67 staining is seen as red nuclear staining.

The performance of CINtec® PLUS was evaluated by comparing the results of dual immunostaining obtained from samples in the SurePathTM collection vials with the samples obtained from the concentration tubes. All the cases were analyzed blindly, without the information of patient’s identification, previous cytology results, HPV test results, follow-up biopsy, or any other relevant data, which could influence the results. Additionally, we also evaluated the diagnostic efficacy of the CINtec® PLUS test in detecting epithelial cell abnormalities in cervico-vaginal samples as compared to cervical cytology and HPV testing.

## RESULTS

A total of 52 cases were included in the study. The age range was 34-74 years.

***Cervical smear cytology: *** Squamous epithelial cell abnormalities in these cases included 18 (34.6%) cases of ASC-US (atypical squamous cell- undetermined significance), 9 (17.3%) cases of LSIL, 11 (21.2%) cases of HSIL and 14 (26.9%) cases of squamous cell carcinoma (SCC). Subsequent cervical biopsies were available in 19 (36.5%) cases. The histopathological diagnoses obtained on follow-up biopsies have been listed in Table 1.

**Table 1:** Correlation of cervical cytology with cervical biopsies (n=19).

**Epithelial cell abnormality**	**No. of cases with follow-up biopsies available**	**Histopathological diagnosis on follow-up cervical biopsies**
**ASC-US**	3/ 18 (16.7%)	Squamous metaplasia; No dysplasia or malignancy (n=3)
**LSIL**	4/9 (44.4%)	LSIL/ CIN 1 in two cases; HSIL/ CIN3 in one case; Squamous metaplasia and no dysplasia in one case
**HSIL**	4/11 (36.4%)	HSIL in 3 cases; SCC in one case
**Squamous cell carcinoma**	8/14 (57.1%)	SCC in all 8 cases

**ASC-US:** Atypical squamous cells of undetermined significance, **LSIL:** Low-grade squamous intraepithelial lesion, **HSIL:** High-grade squamous intraepithelial lesion, **SCC:** Squamous cell carcinoma.

***HPV testing by HC2 assay*** *: *HPV testing was performed by Qiagen Hybrid Capture HC 2 assay in all 52 cases. The values were represented in relative light units (RLUs). A total of 34/52 (65.4%) cases were positive for human Papilloma virus (HPV) by HC2 assay. There were 5 (27.8%) ASC-US, 6 (66.7%) LSIL, 10 (90.9%) HSIL, and 13 (92.8%) cases of SCC positive for HPV.

***CINtec® PLUS testing*** *: *The CINtec® PLUS test was performed on all 52 cases. LBC concentration tubes were used in 25 (48.1%) cases and collection vials with preservative fluid were used in 27 (51.9%) cases. The CINtec® PLUS test was positive in 41/52 (78.8%) cases. The results from LBC concentration tubes and collection vials were comparable with 80% CINtec® PLUS positivity in the samples from LBC concentration tubes and 81.5% CINtec® PLUS positivity from the samples in LBC collection vials. This highlights that both sample types can be used for CINtec® PLUS testing with equal diagnostic efficacy. The CINtec® PLUS test was positive in 41/52 (78.8%) cases with positivity in 11 (61.1%) ASC-US, 6 (66.7%) LSIL, 11 (100%) HSIL, and 13 (92.8%) SCC cases.

**Figure 1:**
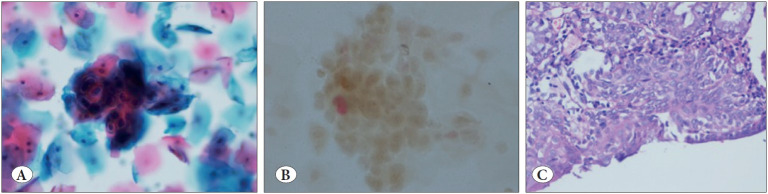
**A)** SurePathTM liquid-based preparation showing squamous epithelial cells reported as atypical squamous cells of undetermined significance (Papanicolaou; x 20). **B)** SurePathTM liquid-based preparation showing negative staining for p16 and Ki-67 by CINtec® PLUS, indicated by the absence of brown cytoplasmic staining and red nuclear staining (CINtec® PLUS; x20). **C)** Section from the same case showing squamous metaplasia without any evidence of dysplasia or malignancy (H&E; x20).

**Figure 2:**
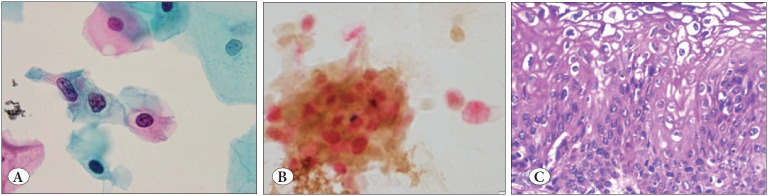
**A)** SurePathTM liquid-based preparation showing scattered koilocytes in a smear reported as low-grade squamous intraepithelial lesion (Papanicolaou; x20). **B)** SurePathTM liquid-based preparation showing positive staining for p16 (brown nucleocytoplasmic staining) and Ki-67 (red nuclear staining) by CINtec® PLUS (CINtec® PLUS ; x20). **C)** Section from the same case showing low-grade squamous intraepithelial lesion with many koilocytes (H&E; x20).

**Figure 3:**
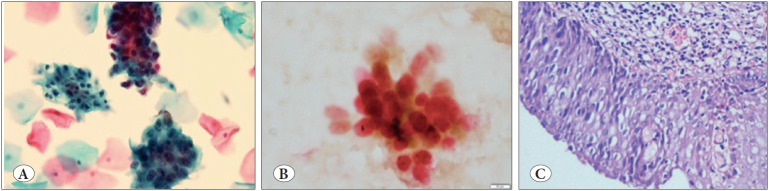
**A)** SurePathTM liquid-based preparation from a case reported as high-grade squamous intraepithelial lesion (Papanicolaou; x20). **B)** SurePathTM liquid-based preparation showing positive staining for p16 (brown nucleocytoplasmic staining) and Ki-67 (red nuclear staining) by CINtec® PLUS (CINtec® PLUS; x20). **C)** Section from the same case showing high-grade squamous intraepithelial lesion with full-thickness dysplasia (H&E; x20).

***Correlation of CINtec® PLUS test results with cervical cytology and tissue biopsies*** *: *Out of 19 cases with follow-up biopsies available, 4 cases (21.1%) (3 reported as ASC-US and 1 reported as LSIL on cytology) did not show any epithelial cell abnormality on follow-up biopsies. Out of 14 SCC cases, 8 were confirmed on follow-up histopathology. Among the cases reported as LSIL on cytology, 1 case was upgraded to CIN3/HSIL and 1 case was reported as squamous metaplasia on subsequent cervical biopsy. One of the cases reported as HSIL on cervical cytology was upgraded as SCC on subsequent histopathology. All the cases reported as ASC-US, with follow-up cervical biopsies (n=3), showed features of chronic cervicitis and squamous metaplasia. No dysplasia or malignancy was noted in these cervical biopsies. On comparing CINtec® PLUS positivity with cervical cytology, CINtec® PLUS positivity was seen in 10 cases of ASC-US, 6 cases of LSIL, 10 cases of HSIL, and 13 cases of SCC (Figure 1 Figure 2 Figure 3).

***Correlation of CINtec® PLUS test results with HPV testing*** *: *On comparing CINtec® PLUS positivity (34/52; 78.8%) with HPV positivity (41/52; 65.4%), dual positivity was seen in three cases of ASC-US, 6 cases of LSIL, 10 cases of HSIL, and 13 cases of SCC (Figure 1 Figure 2 Figure 3). One case each of HSIL and SCC was negative on the HPV test and positive on CINtec® PLUS. Additionally, there was one case of SCC that was positive on HPV test but where CINtec® PLUS immunostaining was negative. Similarly, there were an additional six cases of ASCUS that were positive for the CINtec® PLUS test but negative for the HPV test. The correlation of CINtec® PLUS test results with cervical cytology and HPV testing has been highlighted in Table 2.

**Table 2:** Correlation of CINtec® PLUS test results with cervical cytology and HPV testing.

**Epithelial cell** **abnormality**	**Positive HPV test** **(n=34)**	**Positive CINtec® PLUS test (n=41)**	**Dual positive for CINtec® PLUS test and HPV test**
**ASC-US**	5/18 (27.8%)	11/18 (61.1%)	3/18 (16.7%)
**LSIL**	6/9 (66.7%)	6/9 (66.7%)	6/9 (66.7%)
**HSIL**	10/11 (90.9%)	11/11 (100%)	10/11 (90.9%)
**Squamous cell carcinoma**	13/14 (92.8%)	13/14 (92.8%)	13/14 (92.8%)

**ASC-US:** Atypical squamous cells of undetermined significance, **LSIL:** Low-grade squamous intraepithelial lesion, **HSIL:** High-grade squamous intraepithelial lesion, **HPV:** Human papillomavirus.

***CINtec® PLUS testing using LBC concentration tubes and collection vials: *** The* *CINTec® PLUS test was positive in 20/25 (80%) cases done from the samples in LBC concentration tubes and 22/27 (81.5%) cases done from the samples in LBC collection vials. There was no statistically significant difference in the results obtained from both types of samples (p>0.05). No technical difficulties were encountered during testing from both sample types. The staining intensity, percentage of positive cells and background staining results were comparable with both sample types. This highlights that either the LBC concentration tube or the LBC collection vial can be used for performing CINTec® PLUS testing.

## DISCUSSION

Cervical cancer screening using Pap-stained cervical smears is the most successful cancer screening program launched till date. Use of this screening method has lead to a significant increase in the detection rates of pre-cancerous lesions and a decrease in morbidity and mortality; however, the test has its own set of limitations. Similarly, human papillomavirus (HPV) testing that is being used as an equivalent effective screening strategy also has diagnostic constraints.

Newer biomarkers are hence being proposed to overcome these limitations. Two such biomarkers are p16INK4a and Ki-67. The concurrent expression of a proliferation marker (Ki-67) and a tumor suppressor protein (p16) may increase the specificity of detection of epithelial cell abnormalities in cases wherein the neoplastic process has been initiated (9). Co-expression of p16INK4a and Ki-67 has been found to be of more diagnostic value than either of these markers alone (10).

Nevertheless, cervical cytology still retains a pivotal role in cervical cancer screening, especially in developing countries and resource-limited settings. This is owing to the fact that well-trained cytopathologists can accurately diagnose cervical precancerous lesions on cytology, more so with the routine use of liquid-based preparations like ThinPrep and SurePathTM. Additionally, HPV testing, when used as a stand-alone cervical cancer screening test, has the disadvantage of not being able to evaluate the degree of cytological abnormality for grading of the epithelial lesions. This shortcoming, however, does not hold true for CINtec® PLUS testing, wherein simultaneous evaluation of cytological atypia, for the grading of the epithelial cell abnormality, is possible along with the evaluation of the dual immunostaining.

In a prospective cervical cancer screening study conducted in Wolfsburg, Germany, 427 out of a total of 7,976 women tested positive for HPV when the cervical Pap smear was negative for any abnormalities. These women were managed with repeat testing and colposcopic examinations and cervical biopsies as clinically indicated. The same cases were also tested with CINtec® PLUS. The CINtec® PLUS kit detected 91.9% of the CIN2 cases and 96% of the CIN3 cases. It was shown to have high sensitivity with a very high degree of specificity. In addition, a negative result for the dual staining had a very high negative predictive value of 99.1% (11).

An international collaborative study was conducted in five European countries to assess the sensitivity and specificity of p16/Ki-67 dual-stained cervical cytology for the detection of high-grade CIN (HGCIN) in primary screening and in ASC-US or LSIL triage settings. This study included 27,349 women attending routine cervical screening. The sensitivity of dual-stained cervical cytology for high-grade cervical lesions was significantly higher (90.1%) than Pap smear cytology (66.4%) in screening. Specificity, however, was similar for both tests (95.3% vs. 95.4%, respectively). The sensitivity of HPV testing was 96.4%, but it had a lower specificity of 90.2%, over all ages, as compared to cytology-based tests. In women aged less than 30 years, specificity of dual-stained cytology was 92.3% as compared to 81.4% for HPV testing. In ASC-US and LSIL triage, dual-stained cytology had high sensitivity, while reducing the number of false-positive results by 43% compared to HPV testing (12).

In another cross-sectional study involving the assessment of the p16 and Ki-67 immunocytochemical expression in negative and equivocal (ASC-US) liquid-based cytology samples testing positive for high-risk HPV types with the HC2 assay or polymerase-chain reaction (PCR), it was concluded that a combination of these two markers can be a useful means for management of these women with equivocal cytology (13).

There have been a few previous studies on dual-immunostaining of p16 and Ki-67 by CINtec® PLUS kit in liquid-based cervical cytology samples (5,6,11–19). The majority of these have been conducted on Thin Prep samples and experience with dual-immunostaining by CINtec® PLUS in SurePathTM liquid-based cervicovaginal samples is quite limited. Therefore, a total of 52 SurePathTM LBC samples reported as ‘squamous epithelial cell abnormality’ were included in the present study to evaluate the performance of the CINtec® PLUS test in LBC samples. Epithelial cell abnormalities in these cases included 18 (34.6%) cases of ASC-US, 9 (17.3%) cases of LSIL, 11 (21.2%) cases of HSIL, and 14 (26.9%) cases of SCC. Subsequent cervical biopsies were available in 19 (36.5%) cases. A total of 34/52 (65.4%) cases were positive for HPV by the Hybrid Capture HC2 assay. There were 5/18 cases of ASC-US, 6/9 cases of LSIL, 10/11 cases of HSIL, and 13/14 cases of SCC that tested positive for HPV. The CINtec® PLUS test was performed on all 52 cases.

Both LBC concentration tubes and collection vials were used for the CINtec® PLUS test and the results were comparable with 80% CINtec® PLUS positivity in the samples from LBC concentration tubes and 81.5% CINtec® PLUS positivity from the samples in LBC collection vials. This highlights that both sample types can be used for CINtec® PLUS testing with equal diagnostic efficacy. The CINtec® PLUS test was positive in 41/52 (78.8%) cases with positivity in 61.1% of ASC-US, 66.7% of LSIL, 100% of HSIL and 92.8% of SCC cases. On comparing CINtec® PLUS positivity (78.8%) with HPV positivity (65.4%), dual positivity was seen in three cases of ASC-US, 6 cases of LSIL, 10 cases of HSIL, and 13 cases of SCC. One case of HSIL that was negative on the HPV test was positive on the CINtec® PLUS test, thereby highlighting an added advantage of the CINtec® PLUS test over HPV testing to detect high-grade lesions. The CINtec® PLUS test helps in detecting squamous epithelial cell abnormality in the cervical smear itself, helping in direct correlation with cytomorphology. The CINtec® PLUS test has the capability of detecting even an occasional transformed cell in cases such as ASC-US.

The present study was limited by a small population size and lack of histopathological follow-up in all the cases. Technical difficulties encountered in CINtec® PLUS testing included glycerine mounting of the smears which was not as stable as DPX mounting. The staining faded after a few days; therefore, they had to be interpreted in the fresh state. Another limitation of this test is the cost as compared to routine cervical cytology or HPV testing.

Nevertheless, we believe that this test has the potential of being a highly useful triage tool for indeterminate results on cervical screening (whether by HPV testing or by cervical cytology). The biggest advantage is that the cytopathologist is able to do simultaneous/real-time assessment of cervical cytological abnormalities along with the interpretation of the immmunostaining, unlike in the case of HPV testing.

To conclude, the CINtec® PLUS test helps in detecting precancerous cervical lesions in diagnostically challenging cases or those having indeterminate results with cervical cytology or HPV testing alone, and hence can be widely applied as a triage tool for confirmation of the neoplastic transformation of cervical epithelial cells, after the initial screening protocol.

## CONFLICTS of INTEREST

The authors declare no conflict of interest.

## FUNDING

The study was supported by Intramural Research Funding by the Post Graduate Institute of Medical Education and Research (reference number 71/2-Edu-16/10)
